# Modern low-field MRI

**DOI:** 10.1007/s00256-024-04597-4

**Published:** 2024-02-21

**Authors:** Tobias Pogarell, Rafael Heiss, Rolf Janka, Armin M. Nagel, Michael Uder, Frank W. Roemer

**Affiliations:** 1 Department of Radiology, University Hospital Erlangen, Friedrich-Alexander-Universität Erlangen-Nürnberg, Maximiliansplatz 3, 91054 Erlangen, Germany; 2 Medical Physics in Radiology, German Cancer Research Center (DKFZ), Heidelberg, Germany; 3 Chobanian & Avedisian School of Medicine, Boston University, Boston, MA USA

**Keywords:** MRI, Low-field, Musculoskeletal radiology, Knee, Joints, Field strength, 0.55 Tesla

## Abstract

This narrative review explores recent advancements and applications of modern low-field (≤ 1 Tesla) magnetic resonance imaging (MRI) in musculoskeletal radiology. Historically, high-field MRI systems (1.5 T and 3 T) have been the standard in clinical practice due to superior image resolution and signal-to-noise ratio. However, recent technological advancements in low-field MRI offer promising avenues for musculoskeletal imaging. General principles of low-field MRI systems are being introduced, highlighting their strengths and limitations compared to high-field counterparts. Emphasis is placed on advancements in hardware design, including novel magnet configurations, gradient systems, and radiofrequency coils, which have improved image quality and reduced susceptibility artifacts particularly in musculoskeletal imaging. Different clinical applications of modern low-field MRI in musculoskeletal radiology are being discussed. The diagnostic performance of low-field MRI in diagnosing various musculoskeletal pathologies, such as ligament and tendon injuries, osteoarthritis, and cartilage lesions, is being presented. Moreover, the discussion encompasses the cost-effectiveness and accessibility of low-field MRI systems, making them viable options for imaging centers with limited resources or specific patient populations. From a scientific standpoint, the amount of available data regarding musculoskeletal imaging at low-field strengths is limited and often several decades old. This review will give an insight to the existing literature and summarize our own experiences with a modern low-field MRI system over the last 3 years. In conclusion, the narrative review highlights the potential clinical utility, challenges, and future directions of modern low-field MRI, offering valuable insights for radiologists and healthcare professionals seeking to leverage these advancements in their practice.

## Introduction

When magnetic resonance imaging (MRI) was initially applied to musculoskeletal imaging in the 1980s, the MRI systems used in clinical practice at the time were low-field permanent magnet systems with field strengths between 0.25 T and 1.0 T, which offered poor image quality and low-to-moderate diagnostic performance [1]. Since then, technological advances mainly focused on the improvement of higher field MRI systems with field strengths of 1.5 T and 3 T that represent the large majority of systems that are now being used in our clinical routine worldwide [2]. These systems provide the advantage of superior image quality compared to low-field magnets due to improved contrast- (CNR) and signal-to-noise (SNR), higher resolution, and shorter acquisition times. However, some studies have shown that higher field strength does not necessarily result in an improvement in diagnostic performance, rather the experience of the radiologist reading the image is a deciding factor [3]. In addition, most comparative studies of 1.5 T vs. 3 T in the domain of MSK imaging have shown at best slight superiority of 3-T scanners with “prettier” images not necessarily translating into improved diagnostic performance or therapeutic relevance [4–7]. The market share of low-field MRI devices < 1.5 T declined sharply over the past decades, from about 30% in 2000 to about 5% today [8]. However, such widespread application of high-field systems in musculoskeletal MR imaging today also has some downsides. These include but are not limited to increased maintenance and acquisition costs compared to lower field systems and considerable constructional expenditures to shield the magnetic field and maintain building statics. Additionally, with high-field MRI systems and particularly with ultrahigh-field MRI systems (e.g., 7 T or higher), special attention must be paid regarding safety when examining patients with medical implants [9, 10].

Poor image quality of early low-field systems was primarily a result of suboptimal hardware and software, but not necessarily field strength per se. Technical advances, including but not limited to hardware gradients, receiver coils, pulse sequence design, and reconstruction algorithms, have substantially improved image quality since those early days [1]. Due to advantages in installation, lower acquisition and maintenance costs, and improved image quality compared to previous systems, modern low-field MRI devices bear potential for widespread application in musculoskeletal imaging, particularly in locations without access to high-field systems [2].

At the University Hospital Erlangen, Germany, we have been using a novel 0.55-T MRI system with a superconducting magnet in our clinical routine since November 2020 and have applied it to a wide range of clinical indications and a variety of anatomical locations. Evidence regarding the diagnostic performance of musculoskeletal imaging on such modern low-field devices with state-of-the-art hardware and software is very limited [11]. Most of the publications on musculoskeletal imaging with low-field MRI systems are several decades old, although the aforementioned advancements in low-field software and hardware seem to have led to a recent resurgence of new studies being conducted [12]. This narrative review intends to provide an overview of the existing studies of conventional low-field MRI devices and our own experiences with a modern 0.55-T whole-body MRI in musculoskeletal imaging over the last 3 years.

## Low-field MRI: general considerations

Essentially, two different types of low-field MRI systems are available for musculoskeletal imaging: permanent magnet systems and superconducting systems. Permanent magnets allow for an open configuration and are typically C-shaped scanners. Moreover, portable systems with permanent magnets for dedicated extremity imaging and even bedside evaluation of the brain are available [13, 14]. In 2021, Mazurek et al. reported a proof-of-concept study on the detection of intracerebral hemorrhage in an intensive care or emergency department setting using a 0.064-T bedside portable MRI. Out of 144 examinations, intracerebral hemorrhage was correctly detected in 45 of 56 cases (80.4% sensitivity), and blood-negative (acute ischemic stroke and healthy controls) cases were correctly identified in 85 of 88 cases (96.6% specificity) [14]. Experiences with such portable or bedside MRI systems regarding musculoskeletal pathologies are missing to date.

Advantages of permanent magnets include their longevity and comparatively low power consumption. Disadvantages are necessary complex correction processes (i.e., shimming) to ensure the homogeneity of the magnetic field and heaviness of permanent magnets [8, 15]. Superconducting low-field MRIs in contrast are based on the same concept as high-field scanners. The lower field strength and associated material savings in the design of the magnet reduce operating and acquisition costs [9]. Regardless of the particular scanner design, low magnetic field strengths generally result in reduced SNR and reduced image quality compared to higher field systems when applying the same image acquisition times [16]. Modern software-based image reconstruction techniques such as iterative image reconstruction or artificial intelligence (AI)–based reconstruction methods as well as acceleration techniques like parallel imaging or simultaneous multislice (SMS) bear the potential to increase SNR or reduce measurement time while maintaining image quality. All of these techniques are applicable to any field strength system, including modern low-field devices [9, 17, 18]. These advancements in low-field imaging have resulted in reasonable examination times for diagnostically fully adequate image quality of different anatomies of the musculoskeletal system (commonly performed in 20–30 min per examination). Potential technical advantages of low-field systems over higher field magnets for musculoskeletal imaging include less pronounced susceptibility artifacts near metal implants and at air-tissue interfaces, the latter potentially allowing for better imaging of geometrically challenging regions, such as the cervical spine [1, 19]. A non-negligible disadvantage of MR musculoskeletal imaging at lower field strengths is the narrower absolute difference in the resonance frequencies of fat and water compared to higher field systems. Separating the overlap of these two peaks is a common challenge for conventional low-field scanners. As a result, homogeneous spectral fat saturation often was not possible in the past [20]. Therefore, in low-field imaging, it was usually necessary to resort to other fat suppression sequences such as Dixon or STIR (short TI inversion recovery) or subtraction techniques in the context of contrast administration, which are often inferior to spectral fat saturation in terms of image quality [21]. The latest generation of low-field MRI seems to have overcome this technical limitation. Our initial experience shows very reliable spectral fat suppression in musculoskeletal imaging. This is achieved by optimized radiofrequency (RF) pulses in the 0.55-T scanner we use. Table 1 provides a broad overview of the general advantages and disadvantages of low-field MRI in musculoskeletal imaging.

**Table 1:** Potential advantages and disadvantages of low-field MRI in musculoskeletal imaging

Advantages	Disadvantages
Clinical use	Clinical use
Degenerative and inflammatory pathologies Diagnostics of oncological musculoskeletal pathologies Acute trauma, e.g., fractures, contusions, and ligament injuries in large joints or the spine Imaging of implants and their immediate vicinity, e.g., diagnosing recurrence of soft tissue tumors around tumor prostheses	Potentially insufficient spatial resolution when examining small peripheral joints such as fingers or wrists Resolution of small pathologies, e.g., assessment of chronic knee pain (cartilage, meniscus), traumatic injury following shoulder dislocation and chronic shoulder pain (Bankart and SLAP lesions)
Technical considerations	Technical considerations
Lower susceptibility at air-tissue interfaces or medical implants such as orthopedic hardware Increased magnetic field homogeneity when imaging geometrically challenging regions such as the cervical spine in adipose patients	Low SNR, problematic when encountering small contrast differences in fat saturated sequences Low resolution, e.g., in small anatomical structures Longer acquisition times resulting in potential motion artifacts Difficulties in spectral fat saturation due to diminutive differences in fat and water resonance frequencies
Socio-economic factors	Socio-economic factors
Decreased acquisition cost Decreased maintenance cost Decreased cost per examination Possible increase in general MRI availability	Necessity of selecting patients according to the suspected underlying medical concern, no stand-alone installations Potential re-examination at high-field scanners when results are inconclusive

## Modern low-field MRI: imaging near metal

A wide variety of materials such as metal and metal alloys are used in orthopedic and trauma surgery procedures. Reliable imaging of the adjacent soft tissues is often required in postoperative evaluation and particularly whenever complications are suspected. Prosthesis loosening, prosthesis infection, or local recurrence after resection of soft tissue tumors with bridging osteosynthesis are typical conditions that need to be evaluated. These indications present a particular challenge for MRI due to metals causing signal cancellation, pronounced artifacts, and a reduction of soft tissue contrast in their immediate vicinity [22]. Susceptibility effects such as these are much less pronounced at low-field strengths which results in superior visualization of such conditions compared to higher field systems and particularly for visualization of periprosthetic and periosteosynthetic anatomy [8]. Modern low-field scanners now allow for the application of metal artifact reduction sequences such as slice encoding for metal artifact correction (SEMAC) [18]. Therefore, in some cases, prosthesis infection or tumor recurrence may now be detected with high diagnostic accuracy which was not possible in the past. Current evidence supporting these assumptions is limited [2]. In 2020, Schröder et al. conducted a small feasibility study which examined eight patients with suspected complications after implantation of a knee total arthroplasty using a 0.25-T scanner. A radiologist assessed the fixation of the prosthesis and the surrounding structures (bones, tendons, ligaments, and muscles). MRI findings were compared with computed tomography, clinical examination results, and intraoperative revision findings. In the majority of cases, MRI findings were consistent with clinical examination findings and intraoperative findings, respectively. MRI findings provided comparable results when compared to computed tomography (CT) in all cases [23].

## Spine

Lower back pain is one of the major burdens of healthcare systems worldwide and the leading cause of disability and productivity loss [24]. While imaging for non-traumatic lower back pain is still controversial [25, 26], in a recently (2021) published statement of the American College of Radiology, MRI is suggested as the initial imaging modality for persistent lower back pain over a 6-week period, in patients with cauda equina syndrome and as first-line imaging in suspected tumor disease or infection [27, 28].

**Fig. 1:**
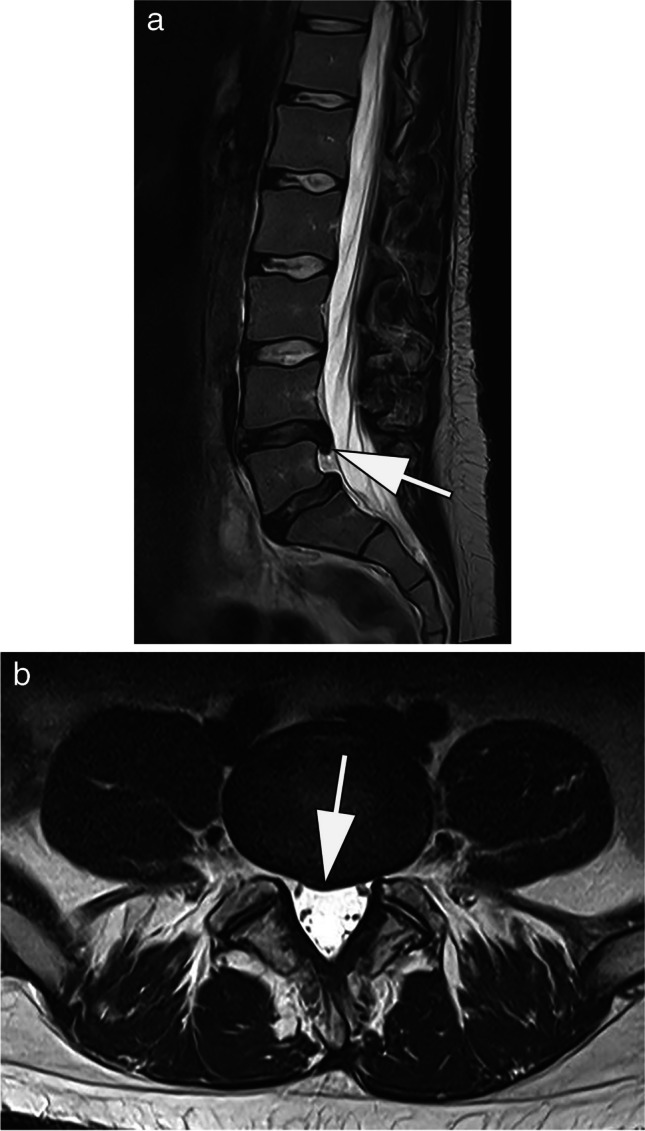
MRI at 0.55 T of the lumbar spine of a 32-year-old patient with non-radiating lower back pain. **A** T2-weighted sagittal image shows a subligamental disc herniation L4/5 inferiorly protruded (arrow). **B** Corresponding axial T2-weighted image at the level of the intervertebral space shows median disc protrusion without recessal obstruction (arrow). Acquisition time (TA): 02:48 min (**A**), 03:35 min (**B**)

Further improvements in the design of receiving coils and the development of multichannel coils offer the prospect of achieving diagnostic quality examinations of the entire spine using low-field MRI [8]. Breit et al. recently assessed the potential of 0.55-T MRI for lumbar spine assessment with and without the use of additional advanced post-processing techniques. Fourteen volunteers were examined at both 0.55 T and 1.5 T using routine clinical sequences, while on the 0.55-T scanner, additional sequences with SMS and AI-based post-processing techniques were acquired. All images were assessed by three radiologists concerning resolution, signal, and contrast as well as visualization of the spinal canal and neuroforamina. While image quality at 0.55 T was perceived inferior to 1.5 T, good overall examination quality and high interrater agreement were reported [27]. In our experience, state-of-the-art low-field MRI is able to reliably answer the main clinical questions including disc herniation and root compression, which are diagnoses of immediate therapeutic relevance in our daily routine (Fig. 1).

## Shoulder

MRI in combination with arthrography is considered to be the gold standard for the evaluation of suspected labral tears or Bankart lesions and infrequently also used for rotator cuff pathology [29]. Direct MR arthrograms have presented a challenge for previous generations of low-field MRI due to the lack of spectral fat suppression commonly applied after intra-articular contrast administration [18]. In an older study (1999), Loew et al. compared MR arthrography of the shoulder at 0.2 T and 1.5 T. For this purpose, 38 patients with chronic instability (*n* = 12) or rotator cuff injury (*n* = 26) were examined at 0.2 T and 1.5 T with MR arthrography. In 27 of the 38 patients, correlation with surgical findings was presented. There was good agreement in the detection of labral injuries and very good agreement in the detection of complete rotator cuff tears between the two scanners. Longer measurement times resulted in an increased risk of motion artifacts, which was considered a disadvantage of the low-field device [30]. In another retrospective study (2014), Lee et al. evaluated the diagnostic accuracy for rotator cuff tears and labral injury at 0.2 T. MR findings from 79 patients were compared with an arthroscopic reference standard within 56 days of MRI imaging on average. This showed good results for the diagnosis of partial (sensitivity of 85% and specificity of 89%) and complete rotator cuff tears (sensitivity of 97% and specificity of 100%). However, superiority for detection of labral anterior and posterior (SLAP) injuries could not be demonstrated (sensitivity of 20% and specificity of 100%) [31].

## Elbow

**Fig. 2:**
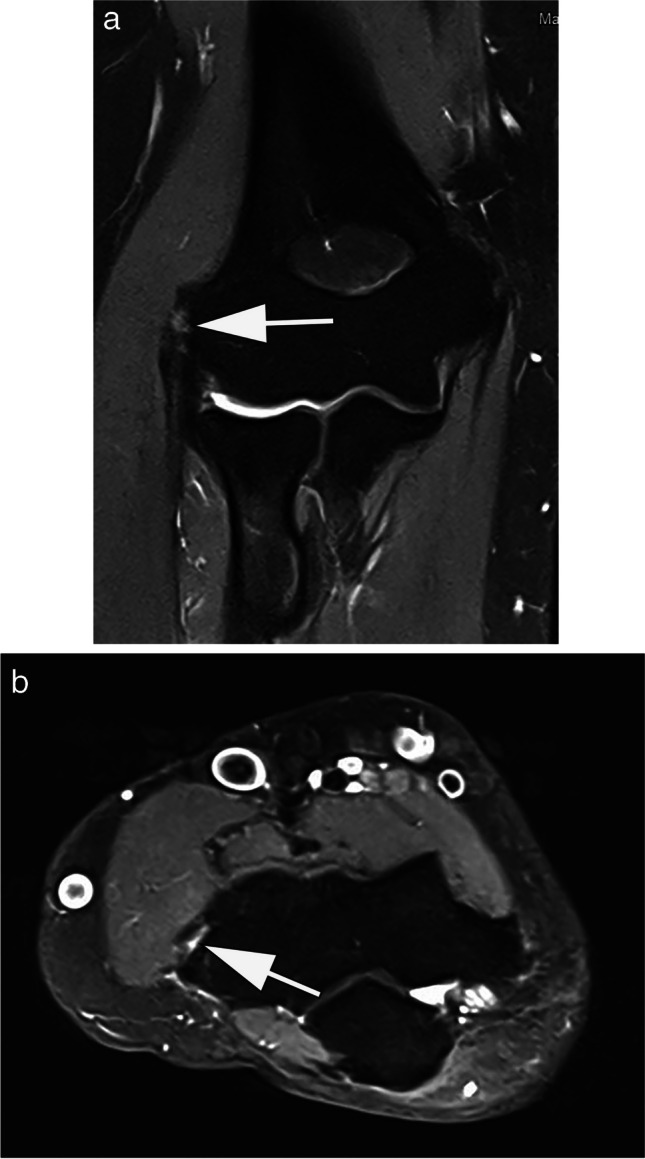
MRI at 0.55 T of the elbow of a 44-year-old patient with lateral elbow pain for the last 4 months. Coronal proton density-weighted image with spectral fat saturation shows increased intratendinous signal at the origin of the common extensor tendon group consistent with epicondylopathy (arrow). **B** Corresponding axial proton density-weighted image shows a small insertional defect consistent with a circumscribed partial rupture (arrow). TA: 01:49 min (**A**), 03:32 min (**B**)

MRI imaging is established for the evaluation of acute and chronic conditions of the elbow joint. Many injuries from acute trauma to chronic repetitive overuse due to sports that put a constant strain on the joint, such as tennis or baseball, involve the ligaments and tendons [32]. In 2015, Okamoto et al. conducted a study that investigated the potential of low-field MRI for the detection of baseball-associated elbow injuries. Asymptomatic baseball-playing children aged 9 to 12 years (*n* = 62) with a history of elbow pain at the time of acquisition were examined with a low-field 0.2-T extremity scanner. Distension and irritation of the ulnar collateral ligament were detected in a large proportion of patients (*n* = 26, 41.9%) [33]. Subsequently, the authors considered the use of low-field MRI as appropriate for early detection of elbow injuries; however, no reference standard related to the pathological confirmation of MRI findings was reported [33]. From our own experience with a 0.55-T MRI, diagnostic quality for assessment of ligamentous injuries of the elbow is adequate as is fracture detection or evaluation of epicondylitis (Fig. 2).

## Wrist

Suspicion of scaphoid fracture is a diagnostic and therapeutic challenge. The fraction of confirmed fractures among suspected diagnoses is low [34]. However, unrecognized and untimely treated scaphoid fractures are commonly associated with complications such as avascular necrosis and the development of non-union [29]. Thus, a highly sensitive and widely available tool for reliable diagnosis of scaphoid fractures is critical. Opposed to the low sensitivity of conventional radiographs in detecting scaphoid fractures, MRI can detect ligament injuries, differentiate occult fractures from bone bruises, and reveal avascular necrosis [29]. In a 2003 study, Brydie et al. examined 195 patients with suspected scaphoid fracture and unremarkable radiographic findings with a 0.2-T permanent magnet extremity scanner. A scaphoid fracture was diagnosed in 37 patients (19%), a fracture of the distal radius in 28 (14%), and fractures of other wrist bones in 9 (5%). In summary, this study showed that low-field MRI was able to establish a definitive diagnosis of wrist fractures or their definite exclusion and subsequently impacted the treatment decision in 92% of cases [35]. Of note, these findings were not compared with other modalities like high-resolution CT.

Without data-supported evidence, from our experience, suitability of low-field MRI for the diagnosis of therapeutically relevant fractures of the carpal bones can also be transferred to modern 0.55-T MRI. However, in contrast to fracture diagnosis, imaging of small anatomical soft tissue structures such as the intrinsic ligaments of the wrist and the triangular fibrocartilaginous complex is likely not suitable in our opinion due to the reduced SNR and should preferably be performed on scanners with higher field strengths and ideally at 3 T [36, 37].

## Hand

Crues et al. reported on the performance of a portable 0.2-T low-field extremity scanner for detecting erosions in the wrist and metacarpophalangeal joints of 132 patients (95% of whom had rheumatoid arthritis) compared to conventional radiographs. Low-field MRI showed a higher proportion of osseous findings with identified erosions in 125 patients (95%) compared to 78 (58%) on conventional radiography [13].

**Fig. 3:**
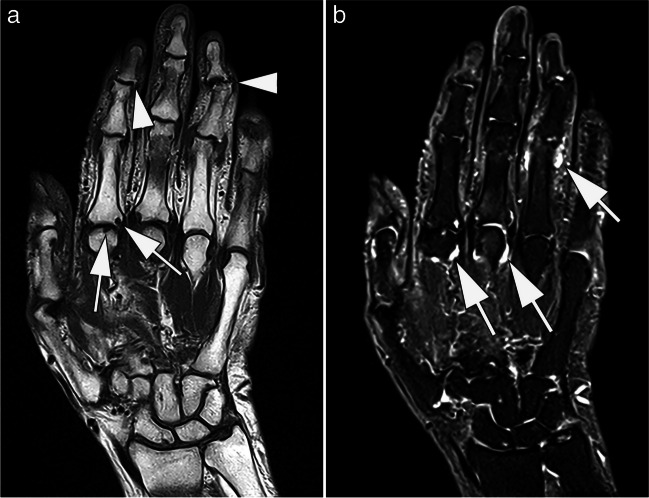
MRI at 0.55 T of the hand of a 77-year old patient with suspected late-onset rheumatoid arthritis. Coronal T1-weighted (**A**) image shows erosions at the 2nd metacarpophalangeal joint (arrows). In addition, osteophytes are depicted at the 2nd and 4th distal interphalangeal joints (arrowheads). **B** Corresponding STIR image shows synovitis at the metacarpophalangeal joints 2 and 3, and at the proximal interphalangeal joint 4 (arrows). TA: 03:20 min (**A**), 05:11 min (**B**)

From our experience, apart from the detection of erosions, modern low-field MRI also allows the detection of other inflammatory joint pathologies such as osteitis, synovitis, and tenosynovitis (Fig. 3). However, additional comparative studies of low-field and high-field systems are mandatory to further evaluate the diagnostic accuracy of low-field MRI in patients with or with suspected rheumatologic diseases, especially with regard to the detection of small erosions.

## Knee

**Fig. 4:**
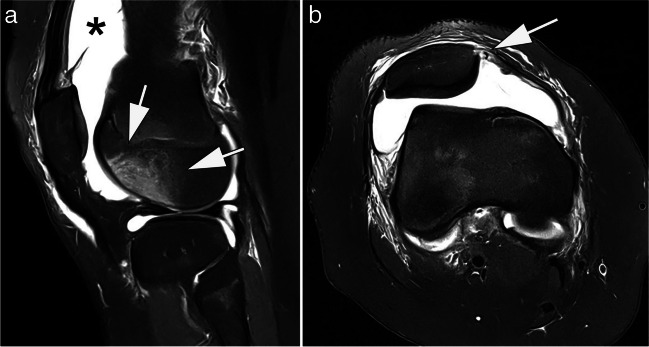
0.55-T MRI of the knee of a 13-year-old girl after knee transient first-time patellar dislocation. **A** Sagittal proton density-weighted image with spectral fat saturation shows a traumatic bone contusion in the anterior-central lateral femoral condyle (arrows) and joint effusion (asterisk). **B** Axial proton density-weighted image with spectral fat saturation shows a rupture of the medial patellofemoral ligament at the patellar attachment (arrow). TA: 04:00 min (**A**), 05:42 min (**B**)

**Fig. 5:**
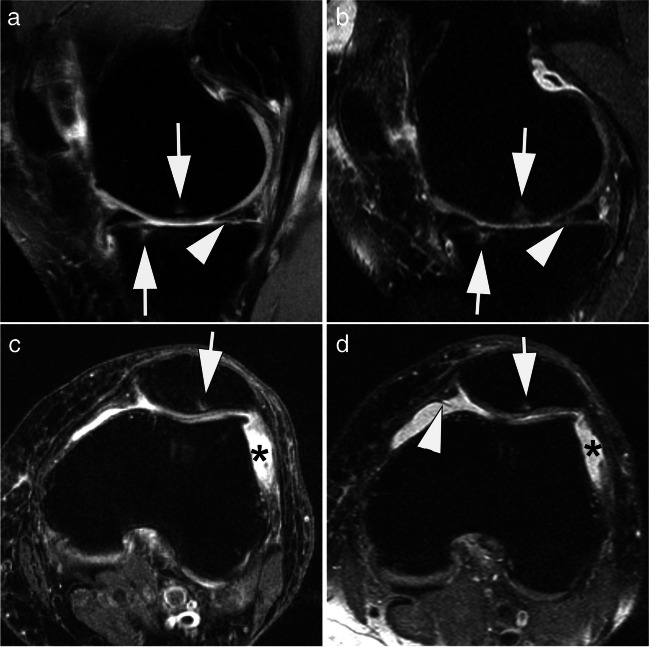
Comparison of a 1.0-T small-bore extremity system with a standard 1.5 T large-bore MRI for assessment of knee osteoarthritis. **A** Standard 1.5-T proton density-weighted fat-suppressed image shows small subchondral bone marrow lesions (BMLs) at the central medial femur and the anterior medial tibia (arrows). In addition, there is a horizontal oblique degenerative tear of the posterior horn of the medial meniscus (arrowhead). **B** At 1.0-T extremity MRI, BMLs (arrows) and tear (arrowhead) are visualized with similar image quality. **C** Axial 1.5-T proton density-weighted fat-suppressed image shows a small BML at the lateral patella (arrow) and joint effusion (asterisk). **D** Axial 1.0-T image shows BML (arrow) and effusion (asterisk) in similar fashion. In addition, there is a small medial patellar plica (arrowhead). TA: 3:53 min (**A**), 4:44 min (**B**), 2:45 min (**C**), 2:59 min (**D**)

MRI is established to diagnose both acute and chronic injuries of the knee [29]. Meniscal and cruciate ligament tears are typical examples of indications for MR imaging of the knee. A few studies have investigated the diagnostic accuracy of low-field versus high-field MRI in meniscal and anterior cruciate ligament (ACL) tears [16]. A study by Vellet et al. (1995) investigated the diagnostic accuracy of ACL tears at 0.5 T and 1.5 T (91%). There was no significant difference in diagnostic accuracy between field strengths [38]. Similarly, in 2000, Cotten et al. found no significant differences in sensitivity and specificity between 0.2-T and 1.5-T MRIs in 90 patients with surgically confirmed meniscal and ACL tears [39]. A 2015 review by Puig et al. also found no difference in diagnostic accuracy for the evaluation of meniscal and ACL tears between low- and high-field MRIs [12], although this was not true for the evaluation of cartilage defects and osteoarthritis due to insufficient data with few studies and small sample sizes [12]. Figure 4 shows an example of acute knee trauma imaged at low-field strength. A dedicated low-field system has been part of the Multicenter Osteoarthritis Study (MOST), a large prospective observational study of knee osteoarthritis (OA) that has been ongoing since 2003. The MRI datasets, based on an optimized sequence protocol [40] and subsequent quantitative and semi-quantitative evaluation acquired within the MOST study, have largely transformed our understanding of OA from a concept of “wear and tear” of cartilage to a whole-joint disorder with multiple tissues involved in the disease process [41–43]. Suitability of the MRI system used (a 1.0-T extremity scanner) for whole-joint assessment of knee OA has been shown in a direct comparison with a standard large-bore 1.5-T system [44]. Comparative image examples are presented in Fig. 5.

## Foot

**Fig. 6:**
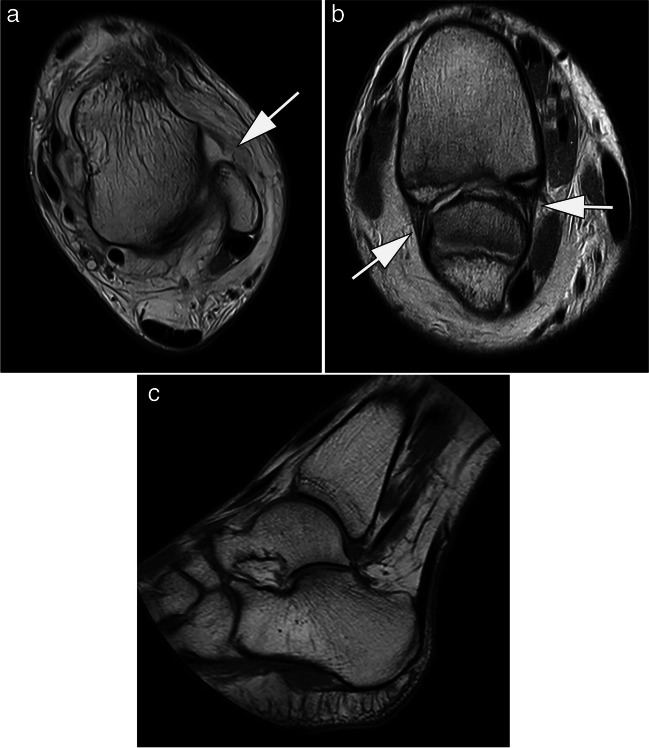
MRI at 0.55 T of the ankle of a 14-year-old patient after ankle sprain. **A** Axial proton density-weighted image shows a complete rupture of the anterior talofibular ligament (arrow). **B** Parasagittal proton density-weighted image demonstrates that the anterior and posterior tibiofibular ligaments are intact (arrows). **C** Small-bore dedicated 1.0-T extremity MRI allows for assessment of the ankle joint, but depending on size of the patient usually plantar flexion is applied as shown in this T1-weighted spin echo image. Note circular field of view of magnet system. TA: 03:07 min (**A**), 05:26 min (**B**), 04:21 min (**C**)

MRI imaging has seen a steady increase in the diagnosis of foot and ankle pathologies [29]. A study published in 1998 found superior image quality of the ankle and foot with a significantly higher SNR in favor of a 1.0-T whole-body scanner compared to a 0.2-T extremity scanner [45]. Despite the differences in image quality, 96% of lesions detected by 1.0-T MRI, such as external ligament injuries or osteochondritis dissecans, were also diagnosed with 0.2 T. However, in a post-examination survey of the included patients on positioning, measurement time, noise, claustrophobia, confidence in the diagnostic procedure, and willingness to repeat the examination, patient acceptance was clearly in favor of the 1.0-T whole-body scanner. Claustrophobia was not a concern for either device; reported noise perception was advantageous for the 0.2-T scanner [45]. In their study from 2000, Herber et al. suggested performing low-field MRI of the ankle joint in children and adolescents with indeterminate ankle pain and unremarkable radiographic findings [46]. Low-field MRI was able to diagnose ligamentous ruptures, fractures, or growth plate injuries in the majority of the 55 patients included, altering therapy regimen in 35 cases [46]. From our own experience with a modern 0.55-T whole-body MRI, acute ankle injuries such as ligamentous ruptures, fractures, and syndesmosis injuries can be diagnosed with high confidence and patient comfort, which needs to be supported by further evidence in the future (Fig. 6).

## Temporomandibular joint

**Fig. 7:**
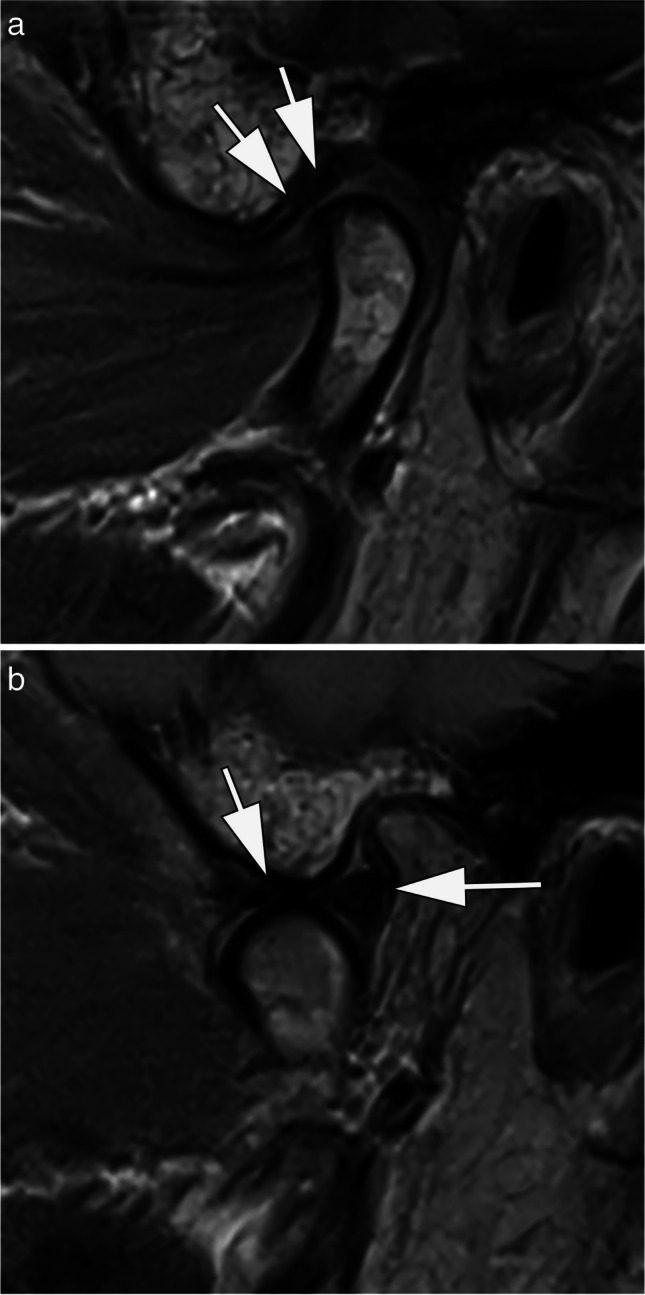
0.55-T MRI of the temporomandibular joint of a 55-year-old patient with chronic pain. **A** Sagittal proton density-weighted image with closed mouth shows a normal disc morphology and position without degenerative changes of the temporomandibular joint (arrows). **B** The open mouth position also shows a normal morphology and position of the disc (arrows). TA: 02:48 min (**A**), 02:48 min (**B**)

Temporomandibular joint disorders are common and have a predominance in young adults and female individuals. Typical symptoms include pain with or without functional limitations such as impaired mouth opening. Due to its excellent soft tissue contrast, high spatial resolution, and the possibility of dynamic examination, MRI is routinely performed in patients with chronic symptoms of the temporomandibular joint. A recent study conducted in 2023 at our institution Kopp et al. compared the image quality at 0.55 T and 1.5 T in 17 patients with a variety of temporomandibular disorders. Both 0.55-T and 1.5-T MRI examinations were performed on the same day. Two senior readers independently evaluated the image quality, graded on a Likert scale and focusing on disc morphology, disc position, and osseous joint morphology. In conclusion, 0.55 T showed overall lower image quality but maintained sufficient diagnostic confidence in the majority of patients (Fig. 7) [47].

## Conclusion

Developments and technical advancements over the past 40 years have led to a redesign of low-field MRI devices [2]. These modern high-performance low-field systems have multiple, previously unexplored clinical applications that have the potential to complement existing high-field diagnostics in musculoskeletal imaging. This has led to a renewed interest to revisit low-field MRI from radiologists and manufacturers alike [18]. Modern low-field MRI systems can provide improved image quality of musculoskeletal structures, particularly at critical air-tissue interfaces and especially with metallic implants. This has to be weighed against the reduced image quality with reduced SNR, reduced resolution, and prolonged measurement times when compared to high-field systems. Lower acquisition and operating costs, as well as potentially portable devices that allow sufficient diagnostic accuracy, may enable more widely available musculoskeletal imaging. Due to the particular specifications of low-field MRI, the patient’s individual medical question and the anatomical structure to be imaged need to be carefully considered before any examination in order to determine whether low-field MRI is an adequate choice (particularly when several field strengths are available). Accordingly, rather than an equivalent or competitive replacement of established high-field scanners, low-field MRI systems should be regarded as an extension of the existing diagnostic portfolio [8]. The currently available evidence regarding the diagnostic performance of modern low-field system for evaluation of the spectrum of musculoskeletal disorders is still limited. The majority of scientific publications are several decades old. The MSK community is encouraged to drive high-quality research to better understand the diagnostic performance, the advantages, and shortcomings of modern low-field MRI systems in musculoskeletal imaging particularly in comparison to higher field strengths in order to fully leverage the potential of these systems.
